# The Dependence of Flue Pipe Airflow Parameters on the Proximity of an Obstacle to the Pipe’s Mouth

**DOI:** 10.3390/s22010010

**Published:** 2021-12-21

**Authors:** Damian Węgrzyn, Piotr Wrzeciono, Alicja Wieczorkowska

**Affiliations:** 1 Polish-Japanese Academy of Information Technology, 02-008 Warsaw, Poland; 2 Institute of Information Technology, Warsaw University of Life Sciences, 02-776 Warsaw, Poland; piotr_wrzeciono@sggw.edu.pl

**Keywords:** flue pipe, organ tuning, pipe’s mouth, obstacle

## Abstract

This paper describes the influence of the presence of an obstacle near the flue pipe’s mouth on the air jet, which directly affects the parameters of the sound generated by the flue pipe. Labial pipes of the most common types of mouth were tested. The method of interval calculus was used instead of invasive measuring instruments. The obtained results prove that the proximity of an obstacle affects the sound’s fundamental frequency, as the airflow speed coming out of the flue pipe’s mouth changes. The relationship between the airflow speed, the value of the Reynolds number, and the Strouhal number was also established. The thesis of the influence of the proximity of an obstacle on the fundamental frequency of the sound of a flue pipe was generalized, and formulas for calculating the untuning of the sound of the pipe were presented for various types of mouth.

## 1. Introduction

Pipe organ tuning is a tedious process. Some of them require many series of tweaks during tuning. Finally, it is possible to tune the instrument. This is one of the reasons why probably no one has analyzed the problems accompanying the tuning process so far.

Another problem is the undoubted lack of time to undertake tests and the lack of willingness to break the routine in the activities performed. Knowledge in the field of organ building is handed down from generation to generation. The next problem is the lack of a sufficient explanation in the scientific papers regarding the everyday work of organbuilders. There are conferences or meetings about organ building but from the musicians’ point of view, not the organbuilders. There are no conferences where the organbuilders could exchange their experiences or discuss together the problems bothering them.

The impetus for this research on the optimization of organ tuning are the problems that have been mentioned by organbuilders for many generations [1,2]. The untuning problem is significant due to its wide range. Two issues suggested the existence of regularities describing the phenomenon of organ untuning. The first one is the situation when the organbuilder tunes the pipes in the workshop on the intonation table, then takes them to the instrument in the destination building, mounts them on the windchest and it turns out that they are not finely tuned. The second issue is in the situation of tuning pipes enclosed in an organ case. The organbuilder finishes tuning the ranks, closes the case and the pipes again are not tuned. This is the issue with small organs, e.g., chest organs.

The problem of untuning is an issue worthy of attention. It is a popular issue as it concerns the work of every organbuilder. Optimizing the tuning process will significantly shorten the time of tuning pipes. This entails savings also in expenditure on the construction or renovation of the instrument, which in the case of the pipe organ is usually one of the key elements of investment. In addition, it will facilitate work with small organs.

In a previous publication [3] the authors have proved that a common element of the tuning process of instruments with pipes densely located on the windchest or closed in a small case is the influence of the proximity of the obstacle to the pipe’s lips. The problem was spotted and experimentally tested on several pipes. The research presented in this paper covers flue pipes of the most common types of mouths and generalizes the thesis of the influence of the proximity of an obstacle on the fundamental frequency of the sound of a pipe. Moreover, the influence of this phenomenon on the parameters of the generated sound was analyzed.

The state-of-the-art research in the area of sound generation in organ pipes raises various aspects, e.g., airflow analysis [4,5,6], or transient state in the process of sound initiation [7,8,9]. The experiments and simulations of sound formation in pipes are reported in literature as well [10,11,12]. There are also works in the field of the acoustics of resonators [13,14,15]. A more detailed review of the state-of-the-art research is presented in Section 7, together with the discussion of our results.

This paper contributes to the research area of sound generated by flue pipes. First of all, it aims to facilitate the process of pipe organ tuning. The results of this research apply to the pipe organ, in which the flue pipes are close to an obstacle. Such an obstacle may be the organ case or the adjacent pipes, which are the obstacles for each other. A good example is a chest organ, where the space on the windchest is limited. We propose an equation that allows determining the fundamental frequency of a pipe’s sound depending on the proximity of an obstacle. This issue is practical and will facilitate the daily work of organbuilders. The novelty is the use of a non-invasive method, with calculations based on the interval calculus.

The paper is organized as follows. Section 2 presents the methodology of measurements and obtaining input data for calculations. Section 3 shows the analysis and processing of the measurement data. Section 4 presents the interval calculus used for further calculations. Section 5 describes the key aspects of sound generation in a labial pipe. The results of the obtained research are presented in Section 6. Section 7 is a discussion and comparison of the results obtained in other works. The outcomes of the research are presented in the Section 8.

## 2. Measurements

**Figure 1:**
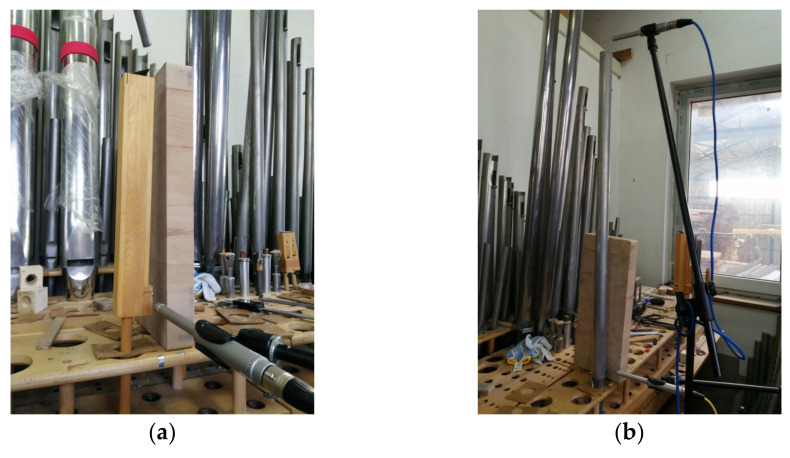
The position of the microphones during recording: (**a**) front view, (**b**) side view.

The measurements of the pipes—both geometric dimensions and acoustic measurements—were carried out on the intonation table in the organbuilder’s workshop. We used two measurement microphones Behringer ECM8000 with omnidirectional characteristics, 1.0 mV/Pa sensitivity, and 20 dBA equivalent noise level. The first of them was used for recording the sound at the top of the pipe (distance from the pipe top about 5 cm), and the second one near the pipe lip (distance from the lip about 5 cm). The sampling rate was 48 kHz. The recordings were repeated at least 5 times. The positioning of the microphones is shown in Figure 1. All photographs in this paper were taken by its authors.

The recordings were made after calibration using an acoustic sound level calibrator Sonopan KA-50 (1 kHz, 94 dB, class 1, IEC 60942:2017 [16], calibration certificate) and a digital sound analyzer Sonopan DSA-50 (class 1, IEC 61672-1:2013 [17], calibration certificate). The Behringer U-Phoria UMC1820 USB audio interface was also used in the sound recording process. Audacity and MATLAB were used as the audio recording software platforms, along with JACK Audio Connection Kit as a sound server. An Asus Zenbook computer with the Intel Core i7 processor, 12 GB of RAM and openSUSE Tumbleweed operating system was used in this work.

An obstacle with an area many times larger than the area of the cut-up was set parallel to the lip and we made 10 measurements at a distance of an integer multiple of 5 mm. The distance of 0 mm means the minimum distance from the lip that could be achieved for a given type of labium (without ears, with ears, with a roller, with a plate, etc.). The analyzed types of pipes with their dimensions, construction and photos are presented in Table 1. The details of the construction of the organ pipe are illustrated in Section 5.

## 3. Data Analysis

In the first step, we selected the central part of each of the audio recordings, representing the steady state. Next, these fragments were transformed into spectra using the Fast Fourier Transform (FFT) with a Hanning window of 65,536 samples. The spectrum calculated via the FFT was used to determine the range in which the fundamental frequency (the first harmonic, usually denoted as *f*_0_) of the sound is located. To determine the precise frequency values, Discrete-time Fourier Transform (DTFT) [18] was calculated in the ranges found with the FFT. The DTFT X(*e^jω^*) for the discrete sequence of the signal x[*n*] is given by Equation (1):

(1) $$X\left(e^{j\omega }\right)=\sum_{n=-\infty }^{\infty }x\left[n\right]e^{-j\omega n}$$

Equation (2) determines the measurement uncertainty *σ_M_* of the obtained results. We used the standard deviation *σ_M_* for individual values of the fundamental frequency.

(2) $$\sigma_{M}=\sqrt{\frac{1}{n-1}\sum_{k=1}^{n}{\left(M\left[k\right]-\mu_{M}\right)}^{2}}$$

where: *µ_M_* is the average of the set M = {M[1], …, M[*n*]}, M[*k*] is the *k*-th value of the M set, *n* is a number of measurements.

The use of DTFT allowed us to determine the fundamental frequency *f*_0_ for each recording with an accuracy of 0.01 Hz, as we calculated DTFT for the frame length equal to the entire sound (about 5 s). The sound level for *f*_0_ was also calculated in relation to the sound level generated by the calibrator and the measurement uncertainty *σ_M_* from Equation (2).

**Figure 2:**
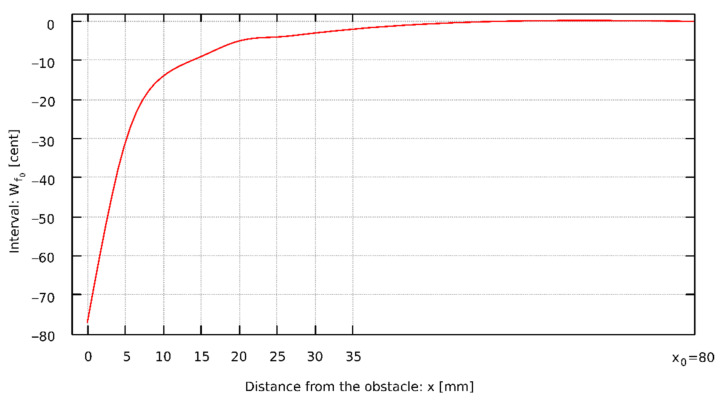
The interval of the fundamental frequency changes in relation to the distance between the obstacle and the pipe lip of Flute 4-foot D sharp.

To describe the changes in sound frequency in the situation of the presence of an obstacle, the intervals W_*f*0_ were calculated, which are expressed in cents and are the musical distances between two sounds. Equation (3) was used to calculate each of the intervals, where *x* is the distance of the obstacle from the pipe lip and *x*_0_ is the distance, at which the sound frequency does not depend on the obstacle. Figure 2 shows the relationship between *f*_0_ and *x*.

(3) $$W_{f_{0}}=1200\log_{2}\left(\frac{f_{0}\left(x\right)}{f_{0}\left(x_{0}\right)}\right)$$

**Figure 3:**
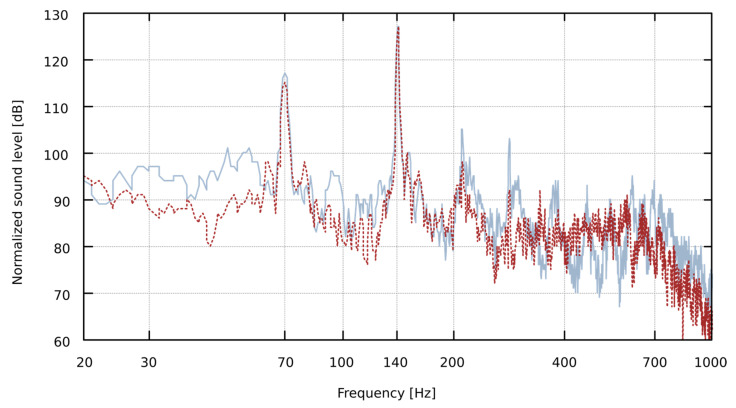
The spectrum of the Bass Principal 16-foot D pipe at the lip (red dotted plot) and the top (grey solid line plot) after normalization to the maximum sound level value for the immediate proximity of the obstacle (0 mm) at the pipe’s lip. As we can see, the harmonics do not overlap. The fundamental frequency, calculated by DTFT with 0.01 Hz frequency resolution, is 69.91 Hz at the lip and 69.69 Hz at the top (a difference of about 5 cents, which can be heard by a trained musician, especially when such a sound is accompanied by another playing pipe).

**Figure 4:**
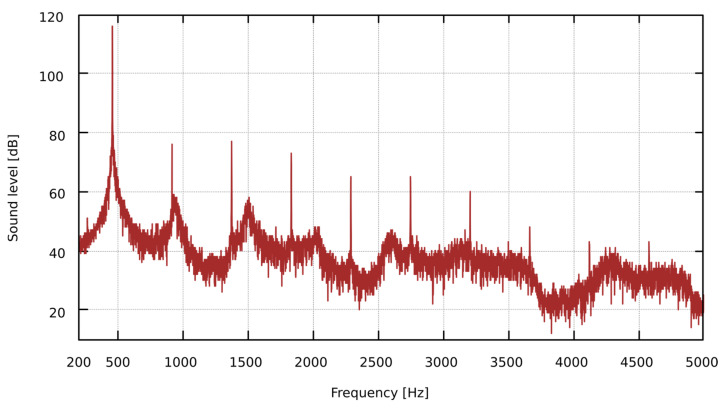
Dolce Flute 4-foot B spectrum for the immediate proximity to the obstacle (0 mm) at the pipe lip. A linear frequency scale has been used to help locate harmonics that are equally spaced on this scale.

Based on the measurements of the behavior of flue pipes, generating sound in the condition of the presence of an obstacle near the labia, we observed the following effects:

- The fundamental frequency of the pipe decreases as the obstacle is closer to the lip. The changes of the pipe sound frequency depending on the distance of the obstacle to the mouth of the pipe are presented in Table 2;
- The spectrum of the sound recorded at the mouth of the pipe may differ significantly from the spectrum of the sound at the top of the resonator (i.a. significant difference in the number of harmonics may be observed);
- The fundamental frequency of the pipe, measured at the top of the pipe resonator, is sometimes different from the fundamental frequency measured at the lip of the pipe. It can be seen in the spectrum, especially on the logarithmic frequency scale, as the harmonics of the sound do not overlap, as shown in Figure 3;
- In the spectrum of some pipes, there are additional components that are not harmonics of the fundamental frequency of the pipe, see Figure 4.

## 4. Interval Calculus

Inserting any measuring instrument inside the pipe disturbs the basic parameters of the sound. The measurement is disturbed also during inserting a measuring instrument between the lip and the obstacle. The same applies to the doping of coloring substances to the air jet in the pipe, as this changes the gas density and its thermal parameters, and thus also distorts the results of the measurements. Consequently, there is a problem with carrying out a direct flow measurement. To solve the impossibility of making the measurement, various mathematical methods to estimate calculations, such as interpolation or approximation, were reviewed.

In this paper, the interval calculus is used to approximate the flue pipe airflow parameters. The use of this method makes it possible to find the data intervals that depend on other known ranges. Interval operations are performed on numerical intervals, and the operation result is also in the interval form. This method began to be developed in the 1960s [19,20] and it is currently used, i.a. to track the uncertainty of various origins, as a tool for finding values that satisfy a specified condition, or as a way of solving nonlinear equations and their systems [21].

This paper uses interval arithmetic based on the operations on intervals [22], presented in Equations (4)–(8).

(4) $$\left[\underline{a},\;\bar{a}\right]\;+\;\left[\underline{b},\;\bar{b}\right]\;=\;\left[\underline{a}+\underline{b},\;\bar{a}+\bar{b}\right]$$

(5) $$\left[\underline{a},\;\bar{a}\right]\;-\;\left[\underline{b},\;\bar{b}\right]\;=\;\left[\underline{a}-\underline{b},\;\bar{a}-\bar{b}\right]$$

(6) $$\left[\underline{a},\;\bar{a}\right]\;\cdot \;\left[\underline{b},\;\bar{b}\right]\;=\;\left[\min\left(\underline{ab},\underline{a}\bar{b},\bar{a}\underline{b},\bar{a}\bar{b}\right),\;\max\left(\underline{ab},\underline{a}\bar{b},\bar{a}\underline{b},\bar{a}\bar{b}\right)\right]$$

(7) $$\left[\underline{a},\;\bar{a}\right]/\left[\underline{b},\;\bar{b}\right]\;=\;\left[\underline{a},\;\bar{a}\right]\;\cdot \;\left[1/\bar{b},\;1/\underline{b}\right],\;0\;\notin \;\left[\underline{b},\;\bar{b}\right]$$

(8) $$⊙\;\in \;\left\{+,\;-,\;\cdot ,\;/\right\},\;a\;\in \;a\;=\;\left[\underline{a},\;\bar{a}\right],\;b\;\in \;b\;=\;\left[\underline{b},\;\bar{b}\right]\;\mathrm{implies}\;a\;⊙\;b\;\in \;a\;⊙\;b$$

where: $\underline{a}$ is the start point of the first interval, $\bar{a}$ is the end point of the first interval, $\underline{b}$ is the start point of the second interval, $\bar{b}$ is the end point of the second interval, ***a***, ***b*** are sets, **min** is the minimum value of the set, **max** is the maximum value of the set.

In the case of interval arithmetic, the result of operations on numbers is contained in the result of operations on intervals. In addition, we used an interval extension of a function, the so-called inclusion function **g** of *g*. It is equivalent to the real-valued function *g*, satisfying the relationship (9).

(9) $$\left\{G(y)\;|\;y\;\in \;y\right\}\;\subseteq \;g(y)$$

where: *y* is an element belonging to the interval, **y** is the interval.

To determine the dependence of some intervals on others we used the natural interval extension, consisting of the same operations as the real-valued function from Equation (9). Additionally, to verify the obtained results, the function h_z_ as the centered form of the inclusion function of h was used [22], satisfying Equation (10).

(10) $$H_{z}\left(y\right)=h\left(z\right)+\left(\nabla h\left(y\right)\cdot \left(y-z\right)\right),\;\mathrm{where}\;z\;\in \;y$$

where: **y** is the interval, *z* is the middle point of **y** interval, ∇h(**y**) is a derivative of h(**y**) function.

## 5. Fundamentals of a Sound Generation in a Labial Pipe

**Figure 5:**
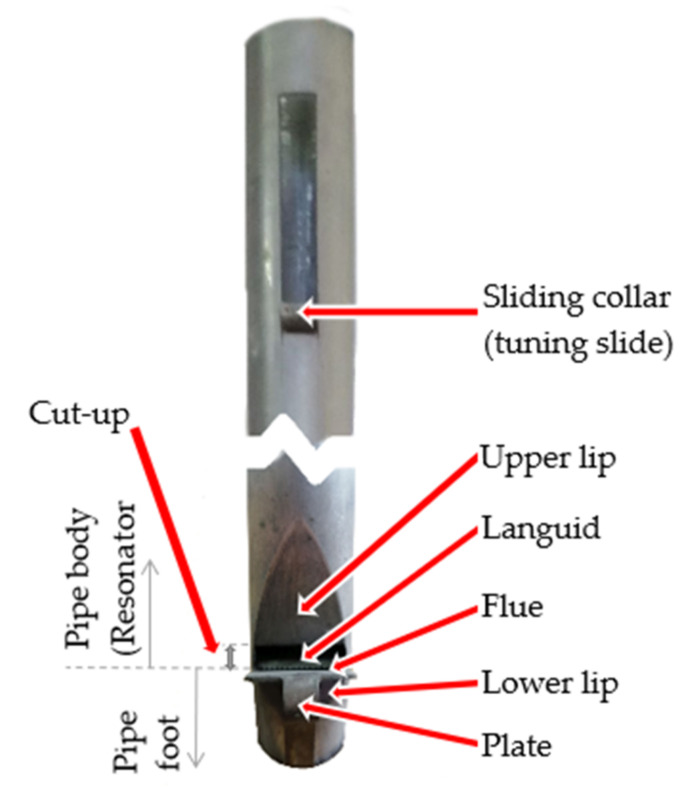
The structure of a flue (labial) pipe.

To generate a sound in a labial pipe, it is necessary to induce turbulent flow [23]. In the pipe, this is achieved by using a very narrow flue in the so-called languid and a wedge cutting the air jet in the form of the upper lip. The wedge angle is usually 30° [24]. The general structure of a flue pipe is shown in Figure 5.

In the basic model of sound generation by a pipe, the phenomena related to turbulent flow are ignored. This model is based on the assumption of an ideal standing plane wave. For such assumptions, we obtain the following dependencies [25]:

(11) $$\lambda_{o}=2l_{p}$$

(12) $$\lambda_{S}=4l_{p}$$

Equation (11) determines the wavelength of the wave generated by an open pipe, where *λ_o_* is the wavelength in an open pipe, and *l_p_* is the length of the resonator. In Formula (12), *λ_s_* is the wavelength for a stopped pipe. The length of the pipe is measured from the lower lip to the end of the sliding collar or the end of the pipe resonator (if there is no sliding collar). 

The frequency of the sound is related to the wavelength through the speed of sound propagation *c*, as shown in Equation (13):

(13) $$f_{pipe}=\frac{c}{\lambda }$$

where: *f_pipe_* is the fundamental frequency of the pipe’s sound [Hz], *c* is the speed of sound [m·s^−1^] and *λ* is the wavelength [m].

The speed of sound *c* in a gas depends primarily on temperature, as shown in Equation (14) [26]. Therefore, the fundamental frequency of the pipe sound must be calculated for a specific temperature, which often differs significantly between the organbuilder’s workshop and the destination place where organ pipes are ultimately assembled.

(14) $$c=\sqrt{\kappa \frac{p}{\rho }}=\sqrt{\frac{C_{p}}{C_{v}}\;\frac{RT}{\mu }}$$

where: *c* is the speed of sound, *κ* is the adiabatic exponent, *p* is static pressure of the gas, *ρ* is gas density, *C_p_* is the specific heat capacity of gas under constant pressure, *C_v_* is the specific heat capacity at constant volume, *R* is a molar gas constant 8.3144621 J·mol^−1^·K^−1^, *T* is temperature [K], *μ* is the molar mass of the gas. For dry air: *κ* = 1.401, *μ* = 0.029 kg·mol^−1^. For the measurement temperature of 19 °C, the speed of sound in the air is *c* ≈ 343 m·s^−1^.

The air jet in a flue pipe is turbulent already in the case of sound initiation, in the so-called initial transient. The vortices are noticeable in the first stages of air jet formation, in the foot of the pipe [27]. At the exit of the air jet from the flue and after reaching the upper lip, significant vortices can be observed in the flowing air mass [28]. The airflow remains turbulent even away from the mouth of the pipe [4]. Contemporary works on the generation of sound in labial pipes use models that take into account the phenomena related to the flow, and we also decided to follow this approach [4,5,27,29]. Expressions (11) and (12) in these papers are only treated as a starting point for further analyzes. 

If there is an obstacle near the pipe’s mouth, there is one more element to consider, namely aerodynamic drag *D* [30], see Equation (15). Approaching the obstacle to the pipe’s mouth increases the area of *S_D_*, drag *D* increases, and velocity *v* decreases.

(15) $$D=C_{D}\frac{\rho \cdot v^{2}}{2}S_{D}$$

where: *ρ* is the fluid density, *v* is the fluid speed in relation to the pipe’s body, *C_D_* is the drag coefficient, *S_D_* is the cross-sectional area of the object exposed to the flow (i.e., the area of the orthographic projection on a plane perpendicular to the direction of the flow).

The drag coefficient *C_D_* depends on the flow velocity, the shape of the streamlined body, and the Reynolds number. The Reynolds number value determines whether the fluid motion is laminar or turbulent. The transition between laminar and turbulent flow occurs at a critical Reynolds number. The critical Reynolds number, below which turbulent flow is not observed, is used in thermodynamics. There is no one, universal value of the critical Reynolds number that assures sound generation [31]. This value is determined empirically depending on the type of flow. The Reynolds number *R_e_* is calculated using Equation (16). For flows in the ranges of large Reynolds number values (over 2000), in which the boundary layer is turbulent, the aerodynamic drag *D* does not change because the drag coefficient *C_D_* is constant [30].

(16) $$R_{e}\;\overset{\mathrm{def}}{=}\;\frac{u\cdot l}{\nu }$$

where: *u* is the flow speed of the fluid (liquid or gas) [m·s^−1^], *l* is the characteristic dimension or length [m], and *ν* is the kinematic viscosity [m^2^·s^−1^].

The characteristic dimension or length *l* concerns the distance, which directly influences the stability of the fluid movement. When the flow is turbulent, the drag coefficient depends only on the Reynolds number, and when it does not change, the drag coefficient does not change either [30,32]. In the pipe there is a turbulent flow, so the drag coefficient *C_D_* can be assumed to be constant. For air at a temperature of about 20 °C, the kinematic viscosity is *ν* = 1.461·10^−5^ m^2^·s^−1^ [33].

Previous measurements and calculations [34,35] have shown that the value of *R_e_* = 2300 is the critical Reynolds number for flows in circular tubes. A flow is always turbulent above that value. In non-circular tube flow systems, the critical Reynolds numbers are different. Moreover, no constant values of critical Reynolds number can be used as they depend on the characteristic dimension or length of various measurable objects. In our case, the characteristic dimension *l* was the distance of the wedge cutting off the air jet in the form of the upper lip from the flue, from which the air is coming out. This selection was based on the papers [7,8,36].

Our work is based on sound generation model taking flow into account [4,5,27,29], as we are interested in flow and fundamental frequency changes. Using the model described in equations from (11) to (14), it is possible to estimate the fundamental frequency of the organ pipe sound, but if there is an obstacle near the pipe’s mouth, the difference between the frequency calculated using this model and the frequency measured from the signal (using the Fourier transform) can be even about 100 cents, i.e., a semitone. In this case, the phenomenon of sound generation by a turbulent flow should also be considered. The generated sound in this context was described by Vincenc Strouhal [37]. He defined the dependence connecting the velocity of the fluid flow *u* (in liquid or gas), the characteristic dimension *l*, and the fundamental frequency of the sound *f*_0_ generated by turbulent flow. This relationship is called the Strouhal number *S_r_* and is described by Equation (17):

(17) $$S_{r}=f_{0}\frac{l}{u}$$

The conditions of the phenomenon described by V. Strouhal are present during the generation of sound in the organ pipe. These conditions are: turbulent flow and the presence of a resonator, in which a certain characteristic dimension *l* can be distinguished [11]. As the air flows, the vortices in the cylinder propagate from opposite sides at a certain fundamental frequency. It results in a fluctuating lift force, depending on the fundamental frequency *f*_0_, and the relationship between them is described by the Strouhal number [12], see Equation (17). In our case, approaching the obstacle to the pipe’s mouth causes the flow velocity changes, and we can use Equation (17) to solve for the fundamental frequency *f*_0_. Therefore, *f*_0_ can be expressed as a function of the Strouhal number.

The relationship between the Reynolds number and the Strouhal number can be determined using Equations (16) and (17), as shown in Equation (18):

(18) $$R_{e}=f_{0}\frac{l^{2}}{S_{r}\cdot \nu }$$

The research on the relationship between the Reynolds number and the Strouhal number [14] shows that the above relationship can also be described by Equation (19):

(19) $$S_{r}=\alpha +\frac{\tau }{\sqrt{R_{e}}}$$

where: *α* and *τ* are constants depending on various intervals of the Reynolds number.

Table 3 presents the values of these constants for the Reynolds number in the situation of exceeding the critical Reynolds number, which is the transition value for turbulent motion. These constants were calculated with the use of linear interpolation by H. Fujita [13].

## 6. Results

### 6.1. Determination of Strouhal and Reynolds Numbers for Labial Pipes

An organ pipe generates a sound only when the airflow is turbulent. Thus, the Reynolds number in this situation exceeds its critical value, hence *R_e_* > 2300 [5]. Additionally, since *f*_0_ >> 1, the Reynolds number is very large. Using Equation (19) and the interval calculus [22], it is possible to determine the range, in which the value of the Strouhal number *S_r_* for the fundamental frequency of the pipe *f*_0_ will fall.

The calculated *S_r_* intervals, depending on the *R_e_* intervals, are presented in Table 4. For example, the *S_r1_* interval, depending on the interval *R_e1_* = [2300, 5000), is obtained as shown below. Using the interval arithmetic for Equation (19) and the values of the constants *α* and *τ* (Table 3) for a corresponding *R_e1_* range, the interval *S_r1_* can be calculated as in Equation (20):

(20) $$S_{r1}=0.2040+\left[\frac{0.3364}{\sqrt{2300}}\right.,\;\left.\frac{0.3364}{\sqrt{5000}}\right)=0.2040+\left(0.048\right.,\;\left.0.007\right]=\left(0.2088\right.,\;\left.0.211\right]$$

The results of calculations presented in Table 4 show that in the case of labial pipes, the expected value of the Strouhal number is in the range [0.1825, 0.211]. Thus, it can be assumed that *S_r_* is approximately constant and the mean for this range is *S_r_* ≈ 0.2. Since the characteristic dimension *l* (the mouth height, called cut-up, see Figure 5) is also constant, then according to Equation (17), the higher the flow velocity *u*, the higher the frequency *f*_0_. As the value of *u* decreases, the frequency *f*_0_ also decreases. Moreover, for the constants *l* and *S_r_* and the known fundamental frequency *f*_0_, the flow velocity *u* can be determined by Equation (17), and then the Reynolds number *R_e_* can be determined from Equation (19), as shown in Table 5.

### 6.2. The Influence of an Obstacle on the Change of Flow Velocity

Let *k* be an integer multiple of 5 mm. We will analyze the influence of an obstacle, placed at distances being integer multiples of 5 mm to the pipe’s mouth, on the fundamental frequency of the generated sound. Based on the Strouhal number analysis in Section 6.1, it can be assumed that the value of *S_r_* is constant. Let *f_k_* be the fundamental frequency of the pipe, calculated by DTFT for the *k*-th distance, and *f*_*k*+1_ for *k* + 1 distance. We denote the airflow velocities in the pipe’s mouth for the *k*-th measurement as *u_k_*, and similarly *u*_*k*+1_ for *k* + 1. Then we have the following dependencies, presented in Equations (21) and (22):

(21) $$f_{k}=S_{r}\;\frac{u_{k}}{l}\;\;,\;\;f_{k+1}=S_{r}\;\frac{u_{k+1}}{l}$$

(22) $$\frac{f_{k}}{f_{k+1}}=\frac{u_{k}}{u_{k+1}}$$

In this case, the change of the Reynolds number *R_e_* from Equation (18) will depend only on the fundamental frequency *f*_0_, which will depend on the flow velocity *u* (Equation (17)).

According to the Kozena-Carman formula [38], for large Reynolds numbers *R_e_* > 4000, the aerodynamic drag coefficient is very small, close to zero [38,39]. Therefore, in the area of influence of the obstacle on the flue pipe sound, the flow velocity should decrease linearly, assuming that the measurement is made every integer multiple of a distance value—in our case by *k* · 5 mm. Therefore, if for each pair (*f_k_*; *f*_*k*+1_) the value of the *f*_*k*+1_/*f_k_* ratio will be similar, then in the flue pipe the velocity of the airflow is directly proportional to the fundamental frequency *f*_0_ of the generated sound.

The performed calculations show that the ratio *f*_*k*+1_/*f_k_* calculated for a single pipe is almost constant and is approximately 1.003. The measurement uncertainty, i.e., the divergence between the calculated and the measured frequency, does not exceed 1% for five-fold measurements of the fundamental frequency of the pipe sound. The smallest dispersion of these proportions is 0.1%. Moreover, calculations from the interval calculus prove to be a good representation of this phenomenon, since the Strouhal number is approximately constant.

### 6.3. The Dependence of the Fundamental Frequency on the Change in the Distance of the Obstacle from the Pipe’s Mouth

**Figure 6:**
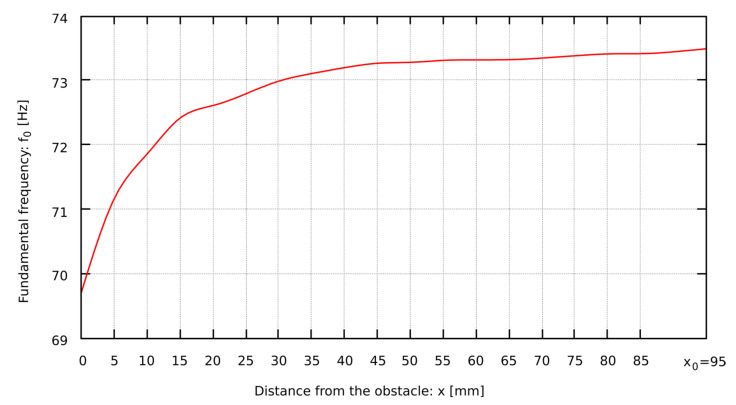
The dependence of the distance of the obstacle from the lips for the Bass Principal 16ft open D.

The relation of the decrease in the fundamental frequency *f*_0_ of the pipe sound as the obstacle is located closer to the mouth of the pipe looks similar for all analyzed types of mouth. The characteristics of this phenomenon are very close to the logarithmic curve (see e.g., Figure 6). Since the occurring phenomena are similar, even though they take place in different geometric conditions, logarithmic regression coefficients were determined according to Equation (23), describing the dependence of the fundamental frequency of the pipe sound on the distance of the obstacle. The data necessary to calculate the sound frequency using Equation (23) for specific pipes are shown in Table 6, i.e., the logarithmic regression coefficients *a* and *b*, the distance *x*_0_ at which the fundamental frequency of the pipe sound is not changed by the obstacle, and the coefficient of determination *r*^2^, that is a measure of the extent to which the model fits into the sample.

(23) $$f_{0}\left(x\right)=a\;\ln\left(\frac{x}{x_{0}}\right)+b,\;\mathrm{for}\;x>0,\;x_{0}>0,\;x\leq x_{0}$$

where: *a*, *b* are the logarithmic regression coefficients, *x* is the distance of the obstacle from the pipe lip and *x*_0_ is the distance, at which the sound frequency does not depend on the obstacle.

Equation (23) was modified by introducing the *b*_2_ coefficient, shown in Equation (24), connecting the *b* coefficient with the fundamental frequency of the pipe *f*_0_. The calculated values of *b*_2_ are presented in Table 6.

(24) $$b_{2}=\frac{b}{f_{0}\left(x_{0}\right)}$$

The *b*_2_ coefficient does not change much. The measurement uncertainty *σ_M_* for different mouth types is 0.001, which is 0.1% of the mean. Thus, it can be assumed that *b*_2_ is constant for all pipes and equals 0.998. This generalization allows using Equation (25) for all pipes in stops, within a specific type of mouth.

(25) $$f_{0}\left(x\right)=a\;\ln\left(\frac{x}{x_{0}}\right)+\left(0.998f_{0}\left(x_{0}\right)\right),\;\mathrm{for}\;x>0,\;x_{0}>0,\;x\leq x_{0}$$

where: *a* is the logarithmic regression coefficient depending on pipe’s mouth type.

## 7. Discussion

One of the assumptions of the presented research was the use of methods that do not interfere with the sound generated in a labial pipe. Research carried out by other authors, to investigate the laws of physics for turbulent air flow, used techniques to color the air, e.g., doping with smoke [6] or inserting the instrumentation inside the pipe [40]. The insertion of the instrument disturbs the basic thermodynamic parameters, which are the basis for calculations and quantitative conclusions. The same problem appears in the situation of introducing coloring pigments that change the parameters of the gas. We were able to carry out the measurements without interfering with the airflow. Thus, the thermodynamic parameters of the air jet remained unchanged. Due to the above measurement limitations, to determine the value of the Strouhal number, we used the interval calculus. It was also not used before in the literature related to the analysis of the pipes.

Czyżewski et al. [11] noted that the ratio of the airflow velocity from the flue *u* to the product of the frequency *f*_0_ and the distance from the upper lip to the flue *l* is constant. Although the authors did not explicitly specify that, this value is the Strouhal number. Hruška and Dlask [7] proved that there is a strong correlation between the Principal Component Analysis component and the reciprocal of the Strouhal number in the initial transient of the pipe sound. Cheong et al. [9], as well as Selfridge et al. [12], confirm the thesis that for Aeolian tone the Strouhal number is constant. In the case of the cylinder, for the fundamental frequency, which dominates the fluctuating lift force, the value of *S_r_* ≈ 0.2 was determined. The stability of the Strouhal number is also confirmed by other researchers [15], analyzing turbulent flow generated by airflow through a flue. They prove that for Reynolds numbers *R_e_* > 2000, the value of the Strouhal number is approximately constant. The authors examining the whistling effect in the tubular system came to similar conclusions [41].

The influence of the obstacle proximity on the sound parameters is often omitted in research. e.g., Odya et al. [10], while carrying out the measurements in the set of pipes, did not consider the too close position of the pipes to each other, which could have a significant influence on the results presented in that paper.

This paper quantitatively determines the phenomenon of changes in the sound parameters of a labial pipe. Such measurement is difficult due to disturbances caused by measuring instruments when they are used close to the pipe’s mouth. This approach has not been used in another study so far. It is worth mentioning that the obtained results are directly dependent on the basic principles of turbulent flow or on the Strouhal phenomenon, which occurs in items similar to the pipes, such as in tubes or slits.

**Figure 7:**
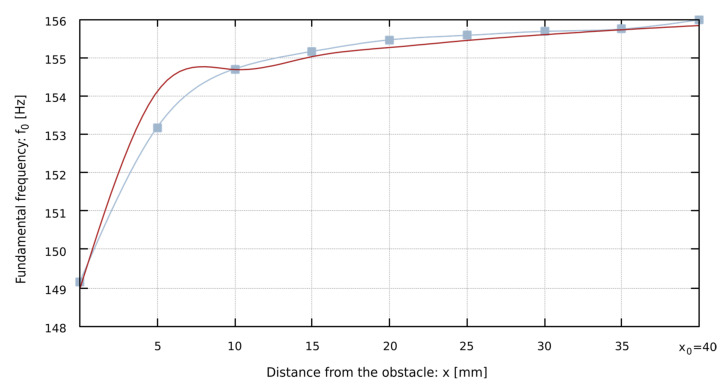
The comparison of the plots representing the dependence of the fundamental frequency *f*_0_ on the distance of the obstacle *x* from the pipe’s mouth for Flute 4ft open D sharp. The grey squares represent the measured data (using DTFT). The grey solid line plot shows the interpolated fundamental frequency for the measured data. The red dotted plot shows data calculated from Equation (25).

It is worth noting that the coefficient of determination *r*^2^ for proposed Equation (25) is 91% on average. It is not an ideal match but this indicates that the function model is sufficiently fitted to the data obtained from measurements and DTFT, which is confirmed by the plots of DTFT values and the values calculated from Equation (25), as shown in Figure 7. Future work may address the issue of developing an improved model of the function *f*_0_(*x*), presented in Equation (25).

## 8. Conclusions

The results of the presented measurements and analyzes of the behavior of the basic parameters of the sound generated by the labial pipe in the presence of an obstacle prove that:

1. The fundamental frequency of the sound is directly proportional to the speed of the airflow in the pipe’s mouth;
2. The speed of the airflow in the pipe’s mouth increases with the distance of the obstacle from the pipe’s mouth;
3. The value of the Reynolds number in the pipe increases with the distance of the obstacle from the pipe’s mouth;
4. The value of the Strouhal number for a labial pipe does not change significantly and can be approximated by a constant value.

The thesis about the dependence of the fundamental frequency of the sound on the distance between the obstacle and the pipe’s mouth was also generalized. The formula describing the untuning of the pipe sound is presented for different types of mouth. The obtained logarithmic regression formulas have a high coefficient of determination, which proves that the models fit the data sufficiently. The proposed Equation (25) can be used in organbuilders’ practice of pipe tuning. The authors believe that this formula is suitable for use in organ building.

The research was carried out in a non-invasive way that does not influence sound parameters, with the use of interval calculus, which has not been used so far in the area of pipe acoustics. Thanks to the use of this method, the obtained conclusions are quantitative.

The conducted research is innovative due to the analysis of the sound parameters of the pipes depending on the obstacles (possibly being other pipes, or organ case) in their surroundings. This is the first study that accurately describes the dependence of the Strouhal number on the fundamental frequency of a sound in the proximity of other pipes or obstacles. Moreover, the use of the method of interval calculus instead of the use of invasive measuring instruments is new. The introduction of measuring equipment significantly disturbs the results because it becomes an additional obstacle for the emitted air jet.

Future work will focus on improving the proposed model for determining the fundamental frequency of sound depending on the proximity of an obstacle. Additionally, interval calculus can be used as a method of determining the ranges for the data inquired for in other research, where invasive measurement methods have been used so far.

## Figures and Tables

**Table 1:** Analyzed flue pipes.

Labium Photo	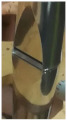	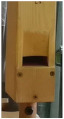	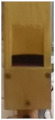	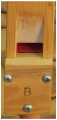	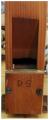	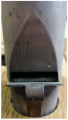	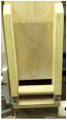	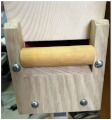
**Labium type**	English bay leaf	Beard	Beard	Beard	Beard	Plate	Roller	Roller
**Stops**	Principal 4ft	Bourdon 8ft	Bourdon 16ft	Dolce Flute 8ft	Flute 4ft	Gamba 8ft	Bass Principal 16ft	Geigen Principal 8ft
**Construction**	Open, pewter (75% tin, 25% lead)	Open, oakwood	Stopped, oakwood	Open, pine	Open, spruce	Open, metal (55% lead, 45% tin)	Open, pine	Open, pine
**Lip width [mm]**	29.4	41.1	41.8	18.5	35.4	29	108	80.5
**Internal pipe dimensions [mm]**	37.9	41.9 × 51.9	42.4 × 54.5	19.4 × 29.2	35 × 51	35.3	106 × 140	79 × 96
**Wavelength [mm]**	530	580	590	290	975	920	2096	2471

**Table 2:** The dependence of the pipe sound frequency on the distance of the obstacle to the mouth of the pipe (the symbol “-” denotes that the pipe generated no sound in this case).

Distance from the Obstacle	Bourdon 16ft Stopped C Sharp	Bourdon 8ft Open B	Principal 4ft E1	Gamba 8ft f Sharp	Geigen Principal 8ft Open C	Bass Principal 16ft Open D	Dolce Flute 8ft Open B	Flute 4ft Open D Sharp
*x* [mm]	*f*_0_ [Hz]							
0	131.44	238.14	-	185.68	64.45	69.69	458.15	149.15
5	135.12	246.02	320.59	186.26	64.80	71.15	464.61	153.18
10	136.95	249.22	324.14	186.46	64.92	71.85	466.35	154.70
15	137.82	250.61	325.28	186.55	65.06	72.41	467.53	155.16
20	138.30	251.30	325.81	186.60	65.16	72.61	468.13	155.46
25	138.60	251.76	325.90		65.17	72.79		155.58
30		251.99	326.16		65.22	72.98		155.68
35		252.19	326.28		65.28	73.10		155.74
40		252.30	326.37		65.31	73.19		
45		252.38	326.44		65.33	73.28		
50			326.49		65.35	73.32		
55			326.52		65.39	73.38		
60					65.39	73.41		
65					65.40	73.41		
70					65.45	73.26		
75						73.31		
80						73.32		
85						73.34		
*x*_0_ (no obstacle)	139.24	252.76	327.19	186.91	65.49	73.49	469.52	155.98

**Table 3:** The values of the constants *α* and *τ*, depending on the intervals of the Reynolds number, used in Equation (19) [13].

***R_e_***	[360, 1300)	[1300, 5000)	[5000, 2 × 10^5^)	[2 × 10^5^, 10^6^)
***α***	0.2257	0.2040	0.1776	0.5760
***τ***	−0.4402	0.3364	2.2023	−175.956

**Table 4:** Interval values of the Strouhal number *S_r_* depending on the intervals of Reynolds number *R_e_* for labial pipes.

***R_e_***	[2300, 5000)	[5000, 10^4^)	[10^4^, 2 × 10^5^)
***S_r_***	(0.2088, 0.211]	(0.1996, 0.2088]	(0.1825, 0.1996]

**Table 5:** Values of Reynolds number *R_e_* and flow velocity *u* for analyzed flue pipes at *S_r_* = 0.2.

Pipe	Bourdon 16ft Stopped c Sharp	Bourdon 8ft Open b	Principal 4ft e1	Gamba 8ft F Sharp	Geigen Principal 8ft Open C	Bass Principal 16ft Open D	Dolce Flute 8ft Open B	Flute 4ft Open D Sharp
***f*_0_ [Hz]**	139.240	252.76	327.2	186.91	65.49	73.49	469.52	155.98
***l* [mm]**	16.53	15.65	7.51	8.1	15	33	8	12.4
***u* [m·s^−1^]**	11.51	19.78	12.29	7.57	4.91	12.13	18.78	9.67
***R_e_***	13021	21187	6315	4197	5043	27389	10284	8208

**Table 6:** Logarithmic regression coefficients and the coefficient of determination for the dependence of the fundamental frequency on the distance between the obstacle and the pipe’s mouth.

Pipe	Bourdon 16ft Stopped c Sharp	Bourdon 8ft Open b	Principal 4ft e1	Gamba 8ft F Sharp	Geigen Principal 8ft Open C	Bass Principal 16ft Open D	Dolce Flute 8ft Open B	Flute 4ft Open D Sharp
***x*_0_ [mm]**	30	50	60	25	75	90	25	40
***a* [Hz]**	0.91	1.76	2.14	0.13	0.12	0.45	1.32	0.83
***b* [Hz]**	138.37	252.50	327.35	186.65	65.36	73.39	468.16	155.83
***b*_2_**	0.994	0.999	1.001	0.999	0.998	0.999	0.997	0.999
***r* ^2^**	91%	96%	85%	88%	84%	90%	95%	97%

## Data Availability

All necessary and relevant data are included in this paper.
